# Occlusion de l'artère de Percheron révélant un lupus érythémateux systémique

**DOI:** 10.11604/pamj.2015.22.30.7760

**Published:** 2015-09-14

**Authors:** Madiha Mahfoudhi, Sami Turki

**Affiliations:** 1 Service de Médecine Interne A, Hôpital Charles Nicolle, Tunis, Tunisie

**Keywords:** Infarctus, thalamus, artère de Percheron, lupus érythémateux systémique, infarct, thalamus, Percheron artery, SLE

## Image en medicine

L'infarctus bithalamique par occlusion de l'artère de Percheron est rare et caractérisé par un retard diagnostique. L'IRM cérébrale et un bilan étiologique bien mené permettent de poser le diagnostic et d'adapter le traitement. Cette complication est exceptionnelle au cours du lupus érythémateux systémique. Patiente âgée de 35 ans hospitalisée pour une perte de connaissance d'installation brutale. L'examen physique a objectivé un érythème en vespertilio du visage, une matité de la base pulmonaire droite, un score de Glasgow à 5 et une hémiparésie droite. Les causes toxicologiques, septiques et métaboliques étaient éliminées. L’électrocardiogramme et l’échographie cardiaque était normaux. La TDM cérébrale initiale était sans anomalie. L'IRM cérébrale a montré des anomalies de signal des deux noyaux thalamiques en hyposignal T1, hypersignal T2 et T2 FLAIR avec restriction de la diffusion en rapport avec un accident vasculaire cérébral ischémique récent bithalamique par occlusion de l'artère de Percheron. Le diagnostic de lupus érythémateux systémique associé à un syndrome des antiphospholipides était retenu devant l'association d'un érythème en vespertilio, une pleurésie, une anémie hémolytique auto-immune avec hémoglobine à 9 g/dl, et un bilan immunologique positif avec présence d'anticorps anti-nucléaires et anticorps anti-DNA à un taux élevé, ainsi que des anticardiolipines fortement positifs à 12 semaines d'intervalle. Le traitement s'est basé sur une corticothérapie à forte dose avec dégression progressive des doses et un traitement anticoagulant. L’évolution était marquée par la disparition des signes cliniques et biologiques.

**Figure 1:**
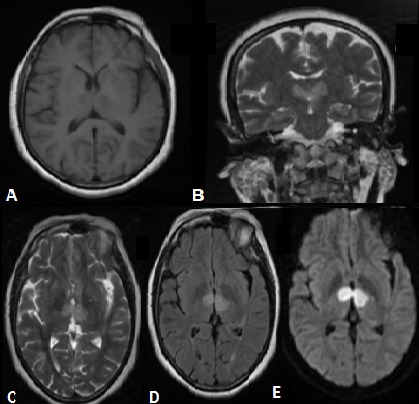
IRM cérébrale en séquences axiales T1 (A),T2 (C), T2 FLAIR(D), diffusion b1000(E) et coronale T2(B): anomalies de signal des deux noyaux thalamiques en hyposignal T1, hypersignal T2 et T2 FLAIR avec restriction de la diffusion en rapport avec un accident vasculaire cérébral ischémique récent bithalamique par occlusion de l'artère de Percheron

